# The Emerging Roles of Metabolite-Activated GPCRs in Teleost Physiology and Aquaculture Development

**DOI:** 10.3390/metabo16010029

**Published:** 2025-12-26

**Authors:** Guan-Yuan Wei, Ming-Yuan Wu, Lan Ding, Zhen-Fa Qin, Zheng-Xiang Zhang, Liang-Jia Wei, Zhi-Shuai Hou

**Affiliations:** 1 Fisheries Research Institute of Fujian, Xiamen 361013, China; 2 China (Guangxi)-ASEAN Key Laboratory of Comprehensive Exploitation and Utilization of Aquatic Germplasm Resources, Ministry of Agriculture and Rural Affairs, Key Laboratory of Aquaculture Genetic and Breeding and Healthy Aquaculture of Guangxi, Guangxi Academy of Fishery Sciences, Nanning 530021, China; 3 Key Laboratory of Mariculture, Ministry of Education, Ocean University of China, Qingdao 266003, China

**Keywords:** metabolite-activated GPCRs, teleost physiology, metabolic signaling, SUCNR1, GPRC6A, GPR142, GPR81, aquaculture development

## Abstract

Metabolites, once viewed mainly as energy substrates or structural precursors, are now increasingly recognized as key extracellular signaling mediators that regulate diverse physiological processes. This review synthesizes and systematizes current knowledge on metabolite-mediated signaling through G-protein-coupled receptors (GPCRs) in teleosts and, importantly, highlights new conceptual links between specific metabolite–GPCR axes and key physiological functions relevant to aquaculture. By integrating evidence across metabolite–GPCRs axes, including succinate–SUCNR1, aromatic amino acids (tryptophan and phenylalanine)–GPR142, basic amino acids (L-arginine)–GPRC6A, and lactate–GPR81. We clarify how metabolite–receptor interactions have the potential to modulate glucose homeostasis, immune responses, energy metabolism, and stress coping. A major contribution of this review is illustrating how metabolites act not only as nutrients but also as extracellular signaling molecules governing core physiological processes via GPCRs. Particularly from an evolutionary perspective, compared with peptide-activated GPCRs, metabolite-sensing GPCRs are relatively conserved across different species, suggesting that relevant findings from biomedical research could be translated to aquaculture applications. Therefore, understanding GPCR-mediated metabolite sensing provides a molecular foundation for improving nutrient formulation, developing functional feeds, and designing selective breeding strategies in precision aquaculture.

## 1. Introduction

GPCRs are evolutionarily conserved membrane proteins mediating versatile signaling, with more than 1000 members identified in humans exhibiting diverse functions [1,2,3]. The A-F classification system is widely used to organize vertebrate and invertebrate GPCRs, which groups GPCRs primarily according to amino acid sequence features and functional similarities [4,5,6]. GPCRs respond to a broad spectrum of extracellular stimuli, including light, ions, odorants, steroids, hormones, neurotransmitters and signaling metabolites [7,8]. Because of the central roles in diverse pathophysiological processes, GPCRs constitute one of the largest families of drug targets. According to a recent publication in Nature Reviews Drug Discovery, approximately 516 approved drugs target GPCRs, accounting for 36% of all approved therapeutics [9]. Compared to humans, teleosts possess an increased number of GPCR-encoding genes due to additional rounds of whole-genome duplication [10,11]. However, studies on GPCRs in economically important teleost species are limited. In the limited studies available on teleost GPCRs, research on the melanocortin receptor family (Class A GPCRs) has demonstrated strong conservation between fish and humans, which includes ligand binding, downstream signaling, and physiological functions [12,13,14,15]. These findings suggest that targeting GPCRs in teleosts holds potential for regulating economic traits of aquaculture species.

Upon ligand binding, GPCRs activate heterotrimeric G-proteins, composed of an α subunit and a βγ dimer, which function as molecular switches for intracellular signal transduction [16]. Ligand-induced conformational changes promote GDP–GTP exchange on the α subunit, leading to its dissociation from the βγ dimer. The activated subunits then regulate downstream effectors via second messengers such as cAMP, IP3, DAG, and Ca^2+^ [17]. The α subunits are classified into four subtypes—Gα_i_, Gα_q_, Gα_s_, or Gα_12/13_—based on structure and function [18,19]. Gα_s_ stimulates adenylyl cyclase to elevate cAMP and activate PKA, whereas Gα_i_ inhibits cAMP production. PKA modulates cytoplasmic, membrane, and nuclear targets, including CREB, thereby mediating both rapid non-genomic and slower genomic signaling [20]. Gα_q_ activates PLC, increasing IP3 and DAG, which trigger Ca^2+^ release from the ER and PKC activation, respectively, regulating metabolism and gene expression. Gα_12/13_ engages RhoGEFs to activate RhoA kinase. Beyond α-subunit signaling, the βγ dimer and other associated proteins, such as β-arrestins, initiate alternative pathways to further regulate cellular responses [21,22,23,24].

Metabolites are traditionally regarded as primary energy substrates or structural precursors within metabolic pathways. Nevertheless, it is well established that numerous metabolites also act as intracellular signaling mediators through interactions with nuclear hormone receptors, including the peroxisome proliferator-activated receptor (PPAR) and the farnesoid X receptor (FXR) [8,25,26]. Noteworthy, accumulating evidence indicates that certain critical metabolites can also function as extracellular signaling mediators [27]. These signaling metabolites originate from dietary nutrients, the gut microbiota, or intermediary metabolism, and they primarily target enteroendocrine, neuronal, and immune cells [8]. Similar to classical neurotransmitters and hormones, these metabolites mainly act through G-protein-coupled receptors (GPCRs) via Gα_i_, Gα_q_, Gα_s_, or Gα_12/13_ protein families [27].

Metabolite-activated GPCRs respond to fatty acids, secondary bile acids, saccharides, lactate, and ketone bodies. Metabolite-activated GPCRs act as sensors of metabolic status and energy availability, helping regulate metabolic hormone secretion or the activity of specific cell types. Many of these receptors are also implicated in the pathophysiology of metabolic disorders, including diabetes, dyslipidemia, and obesity, suggesting that metabolite-activated GPCRs play key roles in regulating body weight, energy homeostasis, and glucose and lipid metabolism [8,28]. These physiological processes are also of significant interest in aquaculture. Teleosts GPCRs are involved in various economic traits and environmental adaptability in fish [12,29,30,31,32]. Teleost GPCRs and specific GPCRs variants exhibit unique pharmacological properties, such as relatively high basal activity [12]. In addition, teleosts have undergone evolutionary genome duplication events, and salmonids and cyprinids in particular experienced a fourth round of whole-genome duplication [10,33]. As a result, certain GPCRs in teleosts have been duplicated, while some key GPCRs are missing [11,33,34]. In peptide-activated GPCRs, evolutionary divergence results in ligands that are not universally applicable between teleosts and humans/mammals. By contrast, metabolite structures are highly conserved across teleosts and mammals, rendering them broadly applicable in aquaculture.

**Figure 1:**
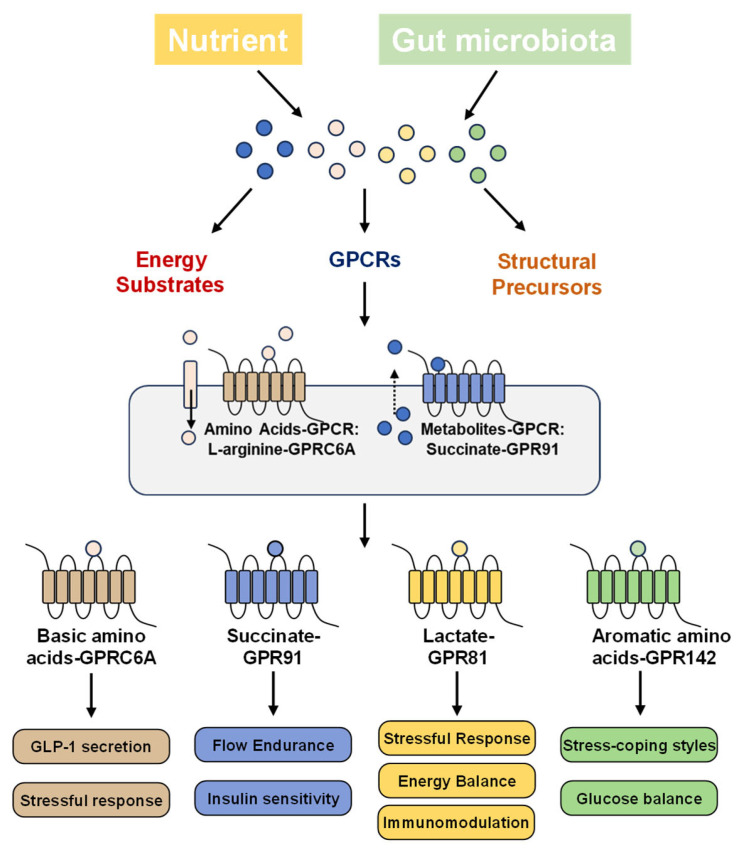
Pathways of metabolite action as nutrients, structural components, and signaling molecules. Metabolites derived from diet or gut microbiota—including succinate, lactate, basic amino acids, and aromatic amino acids—can serve not only as energy or structural substrates but also as ligands for GPCRs, thereby activating their corresponding receptors and potentially regulating multiple aquaculture-relevant physiological mechanisms including stress response, flow endurance, energy balance and metabolism, and immunomodulation.

This review highlights nutrients and metabolites—including succinate, amino acids and lactate—as extracellular signaling molecules with their corresponding GPCRs (Figure 1 and Table 1). Accordingly, this review emphasizes GPCR-mediated metabolic signaling and its physiological implications, providing a foundation for future strategies to target these receptors for genetic improvement, development of functional feeds, and health management in fish farming.

## 2. Succinate and GPR91 (SUCNR1)

### 2.1. GPCR-Mediated Metabolic Signaling of Succinate–SUCNR1 Axis

Among various metabolite-activated pathways, the succinate–SUCNR1 axis represents one of the most thoroughly investigated models in mammals and is an ideal starting point for understanding potential metabolite-driven signaling in teleosts. Succinate functions as a signaling molecule through its cognate receptor, succinate receptor 1 (SUCNR1), a member of the GPCR superfamily also referred to as GPR91 [34,35]. SUCNR1 was regarded as an orphan receptor until its deorphanization in 2004 [36]. Although several other carboxylic acids have been reported to interact with this receptor, their binding occurs with substantially lower affinity and is considered to lack physiological relevance [27]. Under physiological steady-state conditions, circulating succinate concentrations typically range between 2 and 20 μM, but can rise substantially under pathological conditions, reaching up to 100 μM [37,38,39]. Notably, levels within the range of 20–50 μM are sufficient to elicit a half-maximal effective response of SUCNR1, supporting its role as a sensor of homeostatic disturbances [36,37].

Growing evidence indicates that circulating succinate does not derive solely from host metabolism. Gut microbiota-derived succinate has emerged as an additional major source of extracellular succinate and a key regulator of host metabolism and immune responses. In obesity, an elevated ratio of succinate-producing to succinate-consuming bacteria increases circulating succinate, which declines with weight loss [39]. Microbiota-produced succinate supports intestinal gluconeogenesis (IGN) [40], enhances intestinal stem cells (ISCs) activity via mitochondrial energy metabolism [41]. It also promotes pro-inflammatory responses in conditions like inflammatory bowel disease (IBD) through SUCNR1 signaling [42]. These roles highlight the importance of succinate in microbiota–host communication and its potential as a therapeutic target for metabolic and inflammatory diseases. Together, these findings suggest that the microbiota–succinate–SUCNR1 axis may represent a key immune–metabolic regulatory mechanism that extends beyond mammals.

**Figure 2:**
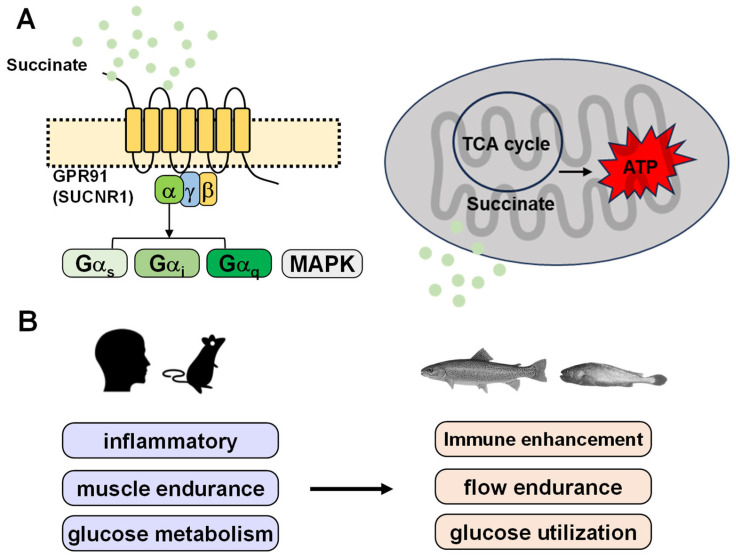
Pathways and physiological functions of succinate. (**A**) Succinate can regulate downstream signaling and physiological functions by activating GPR91, and it also serves as an important circulating metabolite in the tricarboxylic acid (TCA) cycle, contributing to energy production. (**B**) Succinate regulates inflammation, muscle endurance, and glucose metabolism in mammals, showing potential applications in aquaculture.

Biomedical studies showed that SUCNR1 was initially reported to couple with both Gα_i_ and Gα_q_ proteins in human embryonic kidney (HEK293) cells [36]. Subsequent studies consistently confirmed Gi activation, as evidenced by reduced cAMP levels following succinate stimulation (Reviewed in [43]). A recent review highlighted that SUCNR1 couples to heterotrimeric GTPases, specifically Gα_q_, Gα_i_, or Gα_s_ proteins, thereby sustaining the production of diverse intracellular second messengers through cell type-dependent mechanisms (Figure 2A) [35,43,44]. In parallel, succinate–SUCNR1 interaction also activates mitogen-activated protein (MAP) kinases, particularly extracellular signal-regulated kinases 1 and 2 (ERK1/2) [27].

### 2.2. SUCNR1 Regulation of Macrophage Activation, Renal Metabolism, and Tissue Hypoxia Responses in Mammals

Succinate activates SUCNR1 signaling pathways to signal local stress conditions that can influence cellular metabolism. The SUCNR1 is involved in renin-dependent hypertension, ischemia–reperfusion injury, inflammatory and immune responses, platelet aggregation, and retinal angiogenesis. For example, activation of Gα_q_ and Gα_i_ subunits has been linked to pro-inflammatory polarization of myeloid cells [44], whereas Gα_s_ activation is associated with an anti-inflammatory phenotype [45]. In human primary M2 macrophages, extracellular succinate engages SUCNR1 to activate Gα_q_-dependent signaling, thereby regulating immune gene transcription and driving a shift from the M2 to the M1 phenotype [44]. Furthermore, SUCNR1-mediated elevations in blood pressure have been implicated in the progression of diabetic nephropathy and cardiac hypertrophy [43].

### 2.3. Regulatory Roles of the Succinate–GPR91 Axis in Glucose Utilization, Innate Immune and Response to Hypoxia in Teleosts

Although the present insights come primarily from mammalian models, they establish a mechanistic framework useful for interpreting succinate-related functions in teleosts. As an intermediate of the tricarboxylic acid (TCA) cycle [27], succinic acid plays a pivotal role in metabolic regulation and has been shown to promote growth in aquaculture fish species [46,47,48]. For example, dietary supplementation with 0.15% sodium succinate improves glucose homeostasis in zebrafish by enhancing TCA cycle activity [46]. It suppresses intestinal gluconeogenesis, lipolysis, and proteolysis, thereby conserving intestinal protein and lipid [46]. These conserved nutrients are then utilized in the liver and muscle for protein and lipid synthesis, ultimately promoting growth through enhanced energy deposition [46]. In addition, dietary supplementation with 0.02% succinic acid has been associated with improved growth performance, enhanced digestive enzyme activities, and better intestinal development in large yellow croaker (*Larimichthys crocea*) larvae [47]. In largemouth bass (*Micropterus salmoides*), dietary supplementation with succinic acid has been reported to improve starch utilization and growth performance through modulation of the gut–liver axis [48].

Succinic acid has also been reported to enhance innate immune function in aquaculture species. For example, in vitro studies using Nile tilapia (*Oreochromis niloticus*) monocytes/macrophages showed that succinate accumulates significantly following pathogen phagocytosis [49]. Exogenous succinate supplementation dose-dependently increased phagocytic efficiency and upregulated the expression of multiple immune-related genes as well as phagocytosis-related genes. These findings suggest succinate may act as a metabolic cue to upregulate innate immune- and phagocytosis-related genes, thereby facilitating phagocytic activity through the tricarboxylic acid cycle [49]. Similarly, dietary succinic acid has been shown to improve immunity in yellow croaker larvae [47]. Furthermore, studies in Pacific white shrimp (*Litopenaeus vannamei*) demonstrated that succinic acid supplementation increased intestinal short-chain fatty acid content, enriched beneficial microbiota, and enhanced digestive enzyme and immune enzyme activities, along with upregulation of immune-related gene expression [50,51]. Studies investigating GPR91 in teleosts remain scarce. Recent evidence indicates that GPR91 is involved in the hypoxia response mechanisms of fish [52]. Hypoxic stress alters *gpr91* transcription in tiger puffer (*Takifugu rubripes*) [52]. Considering that the succinate–GPR91 axis contributes to regulating cerebral revascularization and tissue restoration following hypoxia–ischemia [53], it is plausible that fish *gpr91* may plays similar roles. It might promote angiogenic processes and preserve vascular density, thereby enhancing oxygen utilization under low-oxygen conditions [52,54].

### 2.4. Aquaculture Relevance: Potential Applications of the Succinate–SUCNR1 Axis in Endurance Exercise, Metabolic Regulation and Immune Responses

Although these findings reveal important physiology of succinate–SUCNR1 in teleosts, knowledge of SUCNR1 remains limited because most studies have focused on dietary supplementation rather than receptor-mediated mechanisms. Therefore, further research is needed to determine whether SUCNR1 is pharmacologically conserved between humans and teleosts, and whether physiological effects of succinate in teleosts act as an energy substrate, a signaling metabolite, or both. Evidence from mammalian studies indicates that succinate levels increase during endurance exercise, with metabolic remodeling of trained muscle characterized by mitochondrial reprogramming and improved systemic insulin sensitivity [55]. In non-myofibrillar cells, SUCNR1 has been demonstrated to participate in transcriptional programs regulating muscle remodeling [56]. Furthermore, dietary succinate-induced activation of SUCNR1 and its downstream Ca^2+^/nuclear factor of activated T cells (NFAT) signaling enhances skeletal muscle endurance capacity in mice [57]. In offshore aquaculture, species such as Atlantic salmon, rainbow trout, and large yellow croaker are frequently exposed to high hydrodynamic environments, where strong water currents impose stress, underscoring the need to improve flow endurance in farmed fish (Figure 2B). Current understanding is largely inferred from mammalian studies, and in teleosts, direct evidence demonstrating whether SUCNR1 participates in the regulation of endurance exercise and muscle remodeling remains limited.

Moreover, carnivorous fish species often exhibit low carbohydrate utilization efficiency [58]. Mammalian models show that conditional inactivation of SUCNR1 elevates insulin levels, impairs glucose tolerance, and increases the proportion of pro-inflammatory macrophages [27,45]. Meanwhile, succinate activates SUCNR1, promoting polarization from the M2 to the M1 macrophage phenotype [44]. However, the role of the succinate–SUCNR1 axis in regulating insulin sensitivity and the shift in macrophage phenotype in teleosts has not yet been functionally validated. Whether SUCNR1 represents an appropriate regulatory target in fish, including the identification of its responsive cell types and downstream signaling pathways, remains to be determined (Figure 2B). Adding another layer of complexity, host–microbiota interactions may influence SUCNR1 signaling via gut microbiota-derived succinate [39,40,41,42], as demonstrated in mammals but still unexplored in fish. Determining whether teleost intestinal microbiota contribute to succinate-dependent immune and metabolic remodeling could provide a mechanistic basis for microbiota-directed functional feeds and precision aquaculture strategies.

Given the widespread tissue distribution of SUCNR1 and its context-dependent signaling, precise characterization of its target tissues (or cells) and corresponding signaling cascades when activated by succinate as a signaling metabolite is essential. Such insights could provide a foundation for precision aquaculture and efficient physiological regulation in aquaculture, particularly with respect to the development of functional feeds and genetic markers for selective breeding.

## 3. Amino Acids and GPCRs

### 3.1. Aromatic Amino Acids and GPR142

#### 3.1.1. GPCR-Mediated Metabolic Signaling of Aromatic Amino Acids–GPR142 Axis

Over time, various protein-derived amino acids have been identified to act through specialized GPCRs as sensing and signaling molecules [8]. GPR142 provides a complementary perspective for understanding nutrient-regulated glucose metabolism and immune responses. Since its cloning in 2006 [59], GPR142 has received relatively limited attention within the academic community [60]. Nevertheless, early large-scale GPCR expression profiling conducted by Regard and colleagues [61] revealed that GPR142 is highly, and almost exclusively, expressed in pancreatic islets. Moreover, knockdown studies in zebrafish demonstrated an association between GPR142 deficiency and an obese phenotype [62]. Subsequent work in 2012 by the Amgen group advanced the pharmacological characterization of GPR142, demonstrating that phenylalanine-related synthetic agonists can activate the receptor and that aromatic amino acids serve as its physiological ligands [63,64]. Indeed, GPR142 has been characterized as a receptor for aromatic amino acids (L-Tryptophan or L-Phenylalanine), with tryptophan identified as the most potent and efficacious ligand while L-phenylalanine exhibits comparatively weaker activity [27,63].

#### 3.1.2. GPR142 Control of Glucose Metabolism and Inflammatory Cytokine Production in Mammals

Functional studies revealed that GPR142 couples predominantly to the Gα_q_ signaling cascade, and tryptophan stimulates insulin release from isolated mouse islets in a glucose-dependent and dose-responsive manner. Furthermore, oral administration of tryptophan in mice enhanced glucose tolerance under oral glucose challenge, underscoring the potential role in nutrient-regulated glycemic control [63]. Meanwhile, in GPR142 knockout mice, LPS-induced production of TNF-α and IL-1β was markedly reduced and pro-inflammatory cytokines have been shown to exert direct regulatory control over GPR142 expression [65,66]. In addition, administration of the GPR142 antagonist CLP-3094 significantly ameliorated arthritis symptoms in mice [66]. Collectively, these findings indicate that GPR142 is implicated in the regulation of inflammatory processes and may modulate pro-inflammatory cytokine signaling. These findings have facilitated the development of synthetic GPR142 ligand (agonists and antagonist) as potential therapeutics for metabolic disorders and chronic inflammatory diseases, with some candidates already progressing to animal studies or phase I clinical evaluation [63,65,67].

#### 3.1.3. Regulatory Roles of the Tryptophan and Phenylalanine–GPR142 Axis in Immune Function, Endocrine and Metabolic Process and HPI Axis Activity in Teleosts

Evidences in mammals provide a theoretical basis for understanding the potential roles of GPR142 in teleosts. Tryptophan and phenylalanine playing a vital role in improving immune function in aquaculture species. Tryptophan strengthens immune function in fish by participating in multiple physiological processes within the neuroendocrine–immune network and playing a critical role in macrophage and lymphocyte functions. In European sea bass (*Dicentrarchus labrax*), both tryptophan deficiency (0%) and excess supplementation exceeding 30% of the optimal requirement increased cortisol levels and impaired immune cell responses following bacterial challenge, ultimately reducing disease resistance [68,69]. Meanwhile, phenylalanine improves intestinal immune function in grass carp by modulating cytokine production through the regulation of NF-κB p65, I-κBα, and TOR gene expression. Phenylalanine influences the transcriptional abundance of tight junction proteins, thereby maintaining epithelial barrier integrity. It also regulates the expression of antioxidant enzyme genes via activation of the Nrf2–Keap1 signaling pathway, underscoring its pivotal role in redox balance and intestinal immune defense [70].

Tryptophan contributes prominently to endocrine and metabolic process. Dietary supplementation with 1% tryptophan has been shown to improve stress resilience and mitigate acute stress responses [71], as evidenced by reductions in serum cortisol and glucose levels under thermal stress [72]. Other studies have indicated that tryptophan can suppress pro-opiomelanocortin levels, thereby regulating ACTH and cortisol, or act as a precursor for serotonin, contributing to the modulation of stress-related hypothalamic–pituitary–interrenal (HPI) axis activity in teleosts [73,74]. Phenylalanine has been shown to promote trypsin, lipase and amylase activities with increased weight gain in grass carp (*Ctenopharyngodon idellus*) [75]. Adequate levels of dietary phenylalanine may involve in altered gene expression related to insulin signaling and key factors in fatty acid synthesis, thus stimulating protein and lipid metabolism along with glycolysis in teleosts [76].

Studies in pufferfish have demonstrated that an additional whole-genome duplication event resulted in the expansion of the *gpr142* gene, giving rise to two paralogs, *gpr142a* and *gpr142b* [60]. However, the physiological functions associated with these subtypes remain largely unexplored. Current research on fish GPR142 has primarily focused on zebrafish, where knockdown of *gpr142a* leads to a reduction in lipid content without causing overt developmental abnormalities [62]. In addition, GPR142 signaling appears to modulate immune and inflammatory processes in teleosts. Dietary supplementation with specific feed additives (alginate oligosaccharides) has been shown to suppress *gpr142* expression, thereby attenuating immune inflammatory responses [77].

#### 3.1.4. Aquaculture Relevance: Potential Applications of the Aromatic Amino Acids–GPR142 Axis for Stress Mitigation and Metabolic Regulation

**Figure 3:**
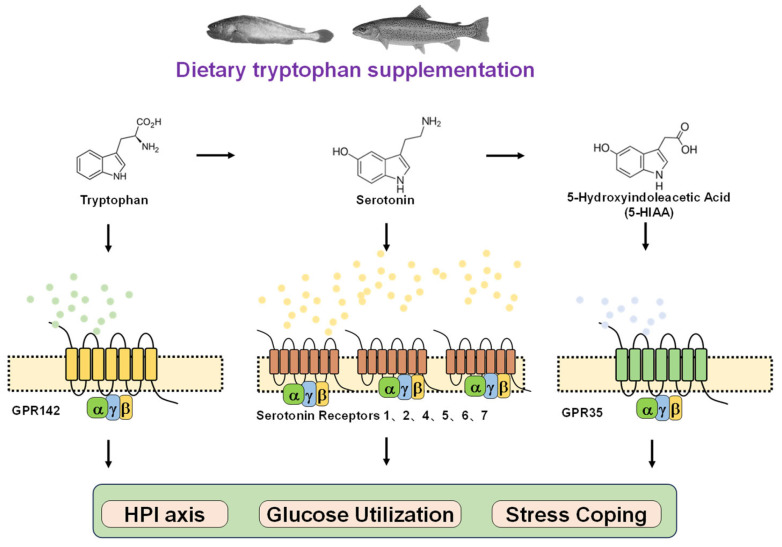
Tryptophan–serotonin pathway with receptors: potential regulators of stress and energy metabolism in teleosts. Tryptophan is a precursor of serotonin. Both are involved in regulating stress responses in teleosts [74,87]. Current research mainly focuses on tryptophan as a precursor of serotonin in modulating teleost stress [87,90,91,92]. Since tryptophan can also activate the receptor GPR142, future studies could further explore the tryptophan and GPR142-dependent pathways in stress response regulation, clarifying the interaction mechanisms among the tryptophan–GPR142, tryptophan–serotonin, and serotonin–serotonin receptor pathways. Similarly, serotonin is further metabolized into 5-hydroxyindole-3-acetic acid (5-HIAA). Both tryptophan and 5-HIAA have regulatory functions in glucose metabolism [63,97]. Activation of the tryptophan receptor (GPR142) has glucose-modulating effects, suggesting that tryptophan might regulate insulin function through GPR142 [67]. Likewise, 5-HIAA also has a corresponding receptor, GPR35. It is therefore worth further investigating the tryptophan–serotonin–5-HIAA metabolic pathway and how each metabolite (tryptophan, serotonin, and 5-HIAA) and its receptor contribute to the regulation of glucose metabolism.

Building on insights from mammalian and teleost studies, these observations provide a scientific rationale for its potential application in aquaculture. Aquaculture species are frequently exposed to diverse environmental stressors, which can lead to reduced feed intake, growth retardation, and in severe cases, mortality, thereby compromising production profitability. In teleosts, stress-coping strategies are generally categorized as proactive and reactive, often described as bold versus shy, or low-response versus high-response phenotypes [78]. Proactive individuals are characterized by specific behavioral and physiological traits, including a greater tendency to explore and take risks in novel environments [79,80], higher feed conversion efficiency, more active feeding behavior after transfer to new conditions [81,82]. Proactive individuals showed reduced sensitivity to environmental stressors [83], lower baseline cortisol levels [84], and attenuated hypothalamic–pituitary–interrenal HPI axis responses under stress [82,85,86]. Studies have demonstrated that brain serotonin levels are associated with stress-coping styles in fish [87]. Proactive rainbow trout exhibit higher baseline serotonergic activity [88], whereas in stress-sensitive Atlantic salmon (*Salmo salar*), cortisol function remains intact but the serotonergic system fails to respond to additional stressors [89]. As the precursor of serotonin, tryptophan has been widely studied and shown to alleviate stress responses in fish [74]. Available teleost studies provide correlative among tryptophan, serotonin and stressful response rather than mechanistic evidence [87,90,91,92]. Most of these investigations in teleosts have emphasized its role as an amino acid energy substrate or serotonin precursor [87,90,91,92], while the physiological functions and signaling pathways of tryptophan as a signaling molecule activating its cognate receptor require further validation (Figure 3).

Recent studies suggested teleosts can be considered as inherently “diabetic-like” organisms, exhibiting pronounced glucose intolerance despite elevated plasma insulin concentrations. Rather than impaired insulin secretion, insufficient peripheral glucose utilization is likely the primary contributor to persistent hyperglycemia in teleosts [93]. Consequently, strategies aimed at enhancing glucose uptake or ameliorating insulin resistance, rather than solely stimulating insulin release with secretagogues such as arginine [94,95], may represent a more effective approach to improving carbohydrate utilization in teleosts. In mammals, oral administration of tryptophan has been shown to enhance glucose tolerance [63]. Although this pathway is well characterized in mammals, its conservation in teleosts has not yet been fully validated. A previous study in blunt snout bream (*Megalobrama amblycephala*) showed dietary tryptophan supplementation improved insulin responsiveness and glucose transport under high-carbohydrate feeding regimes [96]. Whether tryptophan regulates glycemic balance through direct activation of its cognate receptor GPR142 or indirectly as a metabolic intermediate remains unresolved. A recent study demonstrated that the tryptophan-derived metabolite 5-hydroxyindole-3-acetic acid (5-HIAA) mitigates insulin resistance induced by a high-fat diet [97]. Furthermore, synthetic GPR142 agonists have advanced into phase I clinical trials for type 2 diabetes therapy [67]. Therefore, these evidences suggest that tryptophan, acting as a signaling molecule, modulates glucose homeostasis via GPR142, representing a potential regulatory target for improving carbohydrate utilization efficiency in fish (Figure 3).

Nonetheless, current evidence also highlights important knowledge gaps: physiological concentrations of tryptophan in human plasma appear insufficient to activate GPR142 [98,99]. Future research in teleost should therefore clarify whether teleost GPR142 exhibits constitutive activity, determine its EC_50_ and R_max_ values, and characterize the pharmacological and physiological properties of human-derived small-molecule ligands when interacting with fish GPR142.

### 3.2. L-Arginine, L-Lysine and GPRC6A

#### 3.2.1. GPCR-Mediated Metabolic Signaling and Physiology of L-Arginine, L-Lysine–GPRC6A Axis in Mammals

Although GPR142 emphasizes aromatic amino acid sensing, basic amino acids activate a different receptor family with more diverse physiological functions. For example, G-protein-coupled receptor class C group 6 member A (GPRC6A) expands the concept of amino acid-activated GPCRs by linking metabolic regulation with growth, immunity, and inflammatory signaling. GPRC6A is broadly expressed across multiple tissues and organs, including adipose tissue, pancreas, small intestine, skeletal muscle, and brain [100,101,102]. GPRC6A possesses an atypically extended N-terminal domain of approximately 590 amino acids, comprising a Venus flytrap (VFT) module and a cysteine-rich region [103]. Initially deorphanized as an amino acid-sensing receptor, GPRC6A is activated by basic L-amino acids such as L-arginine, L-lysine, and L-ornithine [100,101]. Ca^2+^ and Mg^2+^ serve as positive allosteric modulators that amplify amino acid-induced GPRC6A activation [104,105]. Furthermore, GPRC6A mediates non-genomic signaling in response to the steroid hormone testosterone [106]. The receptor undergoes continuous internalization and is presumed to be recycled through the Rab11-dependent slow trafficking route. This process maintains a stable population of functional receptors on the plasma membrane even under sustained exposure to ubiquitous L-amino acids and divalent cation agonists [107].

Accumulating evidence implicates GPRC6A in multiple physiological and pathological processes, including metabolism and growth, bone homeostasis, inflammation, and prostate tumorigenesis [103,108]. For example, GPRC6A, functioning as one of the amino acid-sensing receptors in the gastrointestinal tract, is proposed to serve as a nutrient sensor regulating feeding behavior. Activation of GPRC6A by amino acids such as L-ornithine promotes glucagon-like peptide-1 (GLP-1) secretion from enteroendocrine L cells, whereas genetic or pharmacological inhibition of the receptor suppresses this effect [109,110]. A prior investigation demonstrated that L-arginine elicited peptide YY (PYY) secretion in both wild-type and GPRC6A-deficient colonic L cells, whereas its capacity to stimulate GLP-1 release was markedly reduced in GPRC6A-deficient cells [111]. Subsequent findings further indicated that GPRC6A activity is not essential for the pronounced in vivo GLP-1 secretion induced by L-amino acids, suggesting the involvement of alternative receptors or compensatory signaling pathways [112]. Notably, other class C GPCRs, such as the calcium-sensing receptor (CaSR) and the T1R1-T1R3, share similar amino acid selectivity and overlapping expression patterns with GPRC6A, supporting their potential contribution to this process [113,114]. Furthermore, GPRC6A functions as a receptor capable of recognizing damage-associated molecular patterns (DAMPs), thereby contributing to sterile inflammation and inflammatory pathologies. Endogenous DAMPs can induce sterile inflammatory responses through G-protein-coupled chemoattractant receptors. GPRC6A and CaSR engage the Gα_q/11_ pathway to activate the NLRP3 inflammasome via endoplasmic reticulum-mediated Ca^2+^ release. Moreover, extracellular Ca^2+^ itself potentially serving as a DAMP that amplifies this process through the CaSR/GPRC6A–phospholipase Cβ (PLCβ) axis [115,116,117]. Taken together, mammalian studies delineate the mechanistic roles of the L-arginine–GPRC6A axis in feeding behavior, metabolism and immunity, serving as a reference point for exploring similar pathways in teleosts.

#### 3.2.2. Regulatory Role of L-Arginine–GPRC6A Axis in Growth Performance, Stress Resistance, Immunomodulation and Energy Sense in Teleosts

Different teleost species possess distinct optimal dietary requirements for arginine, and their growth performance reaches maximum when the dietary arginine content approaches this optimal level. For instance, the arginine requirement of hybrid sturgeon juveniles (*Acipenser schrenckii x Acipenser baerii*) has been estimated at 2.47%. When the dietary arginine level was 1.76%, the specific growth rate (SGR) was significantly lower than that observed in groups fed diets containing 2.64%, 2.93%, or 3.24% arginine (*p* < 0.05). Moreover, the hepatic *gh* and *igf-I* gene expression levels in fish fed 2.64%, 2.93%, 3.24%, and 3.53% arginine were markedly higher than those in fish fed 1.76%, 2.05%, or 2.36% arginine. The highest SGR and expression of *gh* and *igf-I* were recorded at 2.64% dietary arginine [118]. Arginine also enhances the stress resistance of cultured fish. Under ammonia nitrogen stress, yellow catfish (*Pelteobagrus fulvidraco*) juveniles fed a diet containing 2.81% arginine exhibited the lowest cumulative mortality rate, which was significantly lower than that in fish fed 2.44% arginine, indicating that 2.81% dietary arginine effectively improves resistance to ammonia toxicity [119]. Furthermore, arginine strengthens the immune system of aquaculture species. In a nine-week feeding trial with carp (*Cyprinus carpio*), six diets containing 9.8, 12.7, 16.1, 18.5, 21.9, and 24.5 g/kg arginine were tested. After bacterial challenge with *Aeromonas hydrophila*, survival rates were significantly higher in groups fed 16.1, 18.5, and 21.9 g/kg arginine compared with those fed 9.8 or 24.5 g/kg arginine. Collectively, arginine enhances disease resistance primarily by reinforcing cellular defense systems, increasing the levels of humoral immune factors and immunoglobulin M (IgM). Moreover, the upregulation of TOR and 4E-BP mRNA expression in immune tissues may partly explain the arginine-induced improvement in immune capacity and pathogen resistance [120].

The teleost GPRC6A is involved in the gastrointestinal sensing of amino acids. In gastrointestinal tract, GPRC6A can be activated by amino acids, thereby regulating feeding behavior. In rainbow trout, *gprc6a* gene expression in the intestine gradually increases from the anterior to the posterior segments, with the highest expression observed in the hindgut compared to the foregut and midgut. Interestingly, L-proline and L-glutamate significantly regulate *gprc6a* expression, suggesting that, compared with mammals, fish GPRC6A participates in a broader spectrum of amino acid sensing [121]. Further experiments confirmed that *gprc6a* expression in rainbow trout is regulated by a fishmeal aqueous extract, which may contain Ca^2+^ ions, indicating that GPRC6A can be activated/regulated by amino acids or ions to modulate feeding activity in teleosts [122].

#### 3.2.3. Aquaculture Relevance: Potential Applications of the L-Arginine–GPRC6A Axis for Metabolic and Immune Regulation

Several studies indicate the functional roles of L-arginine–GPRC6A axis in teleosts share some similarities with mammals but also exhibit potential species-specific features, highlighting the importance of translating mammalian insights into aquaculture contexts. L-arginine can interact with two major transporter systems: the solute carrier (SLC) transporter superfamily (e.g., SLC7A1, SLC7A2 and SLC7A3) and GPCRs (GPRC6A) [123,124]. From an aquaculture research perspective, it is essential to elucidate whether arginine modulates fish growth, stress resilience, and immune competence primarily through transporter-mediated mechanisms, GPCR-dependent signaling, or an integrated dual regulatory pathway. Considering that GPRC6A recognizes a broad range of endogenous ligands and can couple to multiple G-protein-associated signaling cascades, further investigations are warranted to delineate its tissue-specific expression patterns and downstream pathways in teleosts. Special attention should be directed toward metabolic organs such as the liver and intestine, immune organs including the spleen and head kidney, and reproductive tissues such as the testis. Moreover, the absence of a canonical GLP-1 receptor in teleosts [125] suggests that GLP-1 secretion in teleosts may be regulated through distinct mechanisms. Mammalian studies have demonstrated that arginine stimulates GLP-1 secretion [111]. Functional evidence supporting this mechanism in teleosts is currently lacking. Based on extrapolations from mammalian models, the arginine–GPRC6A axis may represent a potential regulatory target for GLP-1 secretion in teleosts. Meanwhile, contradictory outcomes between mammalian and teleost GLP-1 physiology [125] may stem from the absence of a canonical GLP-1 receptor in teleosts, which likely leads to alternative or species-specific feedback mechanisms and possibly the involvement of non-classical receptors. This divergence highlights the need to investigate other metabolites that may regulate GLP-1 synthesis in teleosts, such as the arginine–GPRC6A axis proposed above. Given that GPRC6A has been identified as a pivotal regulator of inflammatory responses, exploring its role in mediating pathogen- or environment-induced stress in aquaculture species may provide novel insights into the development of GPRC6A-targeted immunostimulants or molecular breeding strategies aimed at enhancing disease resistance and stress tolerance.

## 4. Lactate and GPR81

### 4.1. GPCR-Mediated Metabolic Signaling of Lactate–GPR81 Axis

Beyond amino acid signaling, lactate has emerged as another key metabolic messenger integrating energy status with immune and neuronal regulation via lactate–GPR81 axis. Lactate acts as a bioactive signaling metabolite by engaging the G-protein-coupled receptor GPR81 coupled with Gα_i_-regulated down-regulation of cAMP, mediating its effects through both autocrine and paracrine regulatory pathways [126,127]. GPR81 was first identified in 2001 and subsequently deorphanized in 2008 [128]. GPR81 also belongs to the hydroxycarboxylic acid receptor (HCAR) subfamily, which comprises three homologous receptors: HCAR1 (GPR81), HCAR2 (GPR109A), and HCAR3 (GPR109B) [129]. Early studies on GPR81 primarily focused on white adipose tissue (WAT), where its expression is most prominent [126,130]. Subsequent research has revealed that GPR81 is also expressed in skeletal muscle, the central nervous system, diverse immune cell subsets, and, more recently, in a variety of tumor cell types [127,131].

Emerging evidence from mammalian studies highlights the critical role of microbiota-derived metabolites in host metabolic and immune regulation, suggesting potential conservation of these mechanisms in teleosts. Gut microbiota-derived lactate represents a major source of circulating lactate and plays critical roles in intestinal homeostasis, epithelial repair, and host metabolism. Lactic-acid-producing bacteria (LAB) produce lactate as a key metabolite that promotes intestinal stem cells (ISCs) proliferation and epithelial development through mechanisms involving Wnt/β-catenin signaling and the lactate receptor *Gpr81* [132]. The gut microbiota constitutes the primary source of circulating D-lactate. Oral administration of a biocompatible polymer that sequesters intestinal D-lactate reduced blood glucose and improved insulin sensitivity in obese mice. This intervention also attenuated hepatic inflammation and fibrosis in models of metabolic dysfunction [133]. These findings highlight lactate as a pivotal microbiota-derived metabolite and suggest that the microbiota–lactate–GPR81 axis may have important regulatory roles beyond mammals, providing a rationale for exploring similar mechanisms in teleosts.

### 4.2. GPR81-Regulated Energy Metabolism, Neural Activity, and Inflammatory Regulation in Mammals

Within WAT, GPR81 detects increased lactate concentrations following glucose uptake and subsequently inhibits lipolysis. This regulatory mechanism facilitates the metabolic shift between distinct physiological states, thereby maintaining energy storage under conditions of adequate glucose availability [126,130]. Considering the lactate-rich environment of brain, GPR81-mediated lactate detection has been increasingly recognized as a critical modulatory mechanism operating under both normal physiological states and pathological contexts [27]. GPR81 expression has been reported to increase in ischemic stroke models 24 h following reperfusion, coinciding with a reduction in neuronal cell death. In parallel, lactate acts as a neuromodulator agent by suppressing synaptic activity in both human and rodent brains through GPR81, highlighting this receptor as a potential therapeutic target for epilepsy [134]. Lactate also act as an important regulator of inflammatory processes. Inflammasomes serve as critical signaling hubs for sensing pathogenic microbes and sterile stress signals, culminating in the activation of the potent pro-inflammatory cytokine interleukin (IL)-1β. Toll-like receptors (TLRs) deliver the initial priming signal necessary for inflammasome activation and concurrently promote aerobic glycolysis, leading to lactate production [135]. Lactate binding to GPR81 recruits the intracellular adaptor protein β-arrestin 2, which in turn suppresses NLRP3 inflammasome activation and attenuates the IL-1β-driven pro-inflammatory response. Therefore, GPR81 might serve as a promising immunomodulatory target for inflammatory process [136]. The mammalian studies underscore the versatile role of GPR81 in integrating metabolic, neural, and immune signals, laying the groundwork for examining similar pathways in teleosts.

### 4.3. Regulatory Role of Lactate–GPR81 Axis in Energy Homeostasis, Stress Mitigation and Immune Regulation in Teleosts

Lactate plays a pivotal role in maintaining energy homeostasis in teleost and contributes significantly to their growth performance. Microbiota-derived lactate may further support these processes by promoting gut epithelial function and systemic metabolic regulation, which has been demonstrated in mammalian models [132,133]. In an eight-week feeding trial using three diets containing 0% (control), 1%, and 3% sodium lactate, juvenile Nile tilapia fed the 1% and 3% sodium lactate diets exhibited significantly higher weight gain compared with the control group. Dietary lactate supplementation was shown to suppress both proteolysis and lipolysis, enhance protein and lipid deposition, and facilitate the transformation of lactate into glucose, thereby facilitating improved growth and maintaining overall energy balance [137]. Furthermore, lactate supplementation helps alleviate stress responses and enhances stress resilience in cultured fish. In a 75-day experiment with Nile tilapia, inclusion of an optimal concentration of lactate in the diet mitigated the adverse effects of long-term unchanged water stress, effectively reducing physiological stress markers and improving the capacity to withstand environmental stressors [138]. In addition, lactate has been demonstrated to strengthen the immune system of aquaculture species. Dietary lactate supplementation in rainbow trout improved hematological indices, humoral antioxidant and immunological parameters [139]. It also positively modulated the intestinal microbiota composition, collectively indicating an overall improvement in systemic immune competence and gut health [139].

Current knowledge of GPR81 function in teleosts is relatively limited. Similar to *gpr142*, zebrafish possess duplicated *gpr81* genes, giving rise to two subtypes, both of which can be activated by lactate [140]. In mammals, lactate enhances GPR81 expression and exerts a lipolytic-suppressing effect through receptor expression [141,142]. Interestingly, studies in rainbow trout later revealed that lactate does not alter *gpr81* transcription [143]. One possibility is that although lactate does not increase *gpr81* transcription, extracellular lactate concentrations may not be sufficient to saturate GPR81 binding. A proportion of spare receptors may buffer this effect (spare receptor indicates a full biological effect can be achieved when only a small percentage of receptors are engaged by the ligand [144,145]). In addition, lactate may enhance downstream GPR81 signaling pathways without altering its mRNA levels. Therefore, further experiments are required to assess GPR81 cell surface protein expression and intracellular signaling activity. Such studies remain largely unexplored in teleosts, which is one of the motivations for this review.

**Table 1:** Ligand characteristics, signaling mechanisms, and known or proposed physiological functions of each metabolite–GPCR axis.

GPCR	Ligand Affinity/Characteristics	Major Signaling Pathways	Reported Functions	Proposed Teleost Functions
**GPR91**	EC50: 20–50 μM in mammals	Gαi, Gαq, Gαs, ERK1/2	Succinate: promote growth [47,48]	Flow endurance [56,57]
			Succinate: improve glucose homeostasis and starch utilization [46,48]	Systemic insulin sensitivity [55]
			Succinate: enhance innate immune function [49]	
			Succinate: enriched beneficial microbiota and digestive enzyme (pacific white shrimp) [50,51]	
			GPR91: hypoxia-related oxygen utilization [52]	
**GPR142**	Trp: most potent agonist;	Mainly Gαq	Phenylalanine: promote growth [75]	stress-coping styles [74,87,88,89]
	Phe: weaker agonist;		Tryptophan and phenylalanine: improve stress resistance [71,72,73,74]	glucose balance [63,96]
	CLP-3094: antagonist		Tryptophan, phenylalanine and GPR142: improve immune function [68,69,70,77]	
			GPR142: Regulate lipid metabolism [62]	
**GPRC6A**	Basic amino acids: L-arginine, L-lysine, and L-ornithine;	Amino acids activate Gαq	L-arginine and GPRC6A: nutrient sensor and feeding behavior [118,121]	GLP-1 secretion [111,125]
	allosteric modulators: Ca2+/Mg2+		L-arginine: growth performance [118]	pathogen- or environment-induced stress [115,116,117,119,120]
			L-arginine: stress resistance [119]	
			L-arginine: immune responses [120]	
**GPR81**	Physiological lactate concentrations	Gαi	Lactate and GPR81: modulate metabolism [132,133,137,143]	stress responses [138]
			Lactate:alleviate stress responses [138]	energy balance [126,130,132,133,137,143]
			Lactate: regulator of inflammatory processes [139]	Immunomodulation [135,136,139]

### 4.4. Aquaculture Relevance: Potential Applications of the Lactate–GPR81 Axis in Immune Modulation

Future studies in aquaculture species should focus on the physiological and molecular mechanisms by which lactate regulates teleost growth, stress responses, and immunity from the perspectives of energy balance and metabolic signaling pathways. This study will provide a foundation for further exploration of GPR81-targeted nutritional, stressful or immunomodulatory interventions. In addition, the lactate receptor GPR81 and the L-arginine and L-lysine receptor GPRC6A are both involved in regulating the physiological functions of the NLRP3 inflammasome [115,116,117,136]. Although numerous studies have demonstrated that NLRP3 plays a crucial role in restricting bacterial infections and mediating anti-infection immune defense in teleosts, studies on the use of exogenous regulators of teleost NLRP3 remains limited. Based on findings from mammalian models, future studies could further investigate the regulatory effects of the lactate–GPR81 and basic amino acids–GPRC6A axes on NLRP3 in teleosts.

## 5. Key Knowledge Gaps in Teleost Metabolite-Sensing GPCRs

Despite growing evidence for the importance of metabolite-activated GPCRs, functional studies in teleosts remain limited. The main key gaps include the following aspects.

Numerous studies have assessed the dietary metabolite requirements of cultured fish species, such as salmonids, tilapia, and carp. For example, studies in rainbow trout and common carp have shown that approximately 5 g/kg of lactate supplementation yields optimal effects, and the optimal tryptophan requirement for rainbow trout and Nile tilapia is around 3.0–4.0 g/kg [139,146,147,148]. However, these studies have primarily focused on metabolites as energy substrates, while their optimal levels as signaling molecules remain to be further investigated.

The pharmacology of receptors such as SUCNR1, GPR142, GPRC6A, and GPR81, for which parameters like binding affinity, EC_50_, constitutive activity, and signaling bias are largely unknown.

The cell type specificity of these GPCRs in immune, metabolic, and endocrine tissues has not been systematically investigated. Meanwhile, most available studies have employed mammalian cell lines, such as HEK293 cells, rather than teleost-derived cells or teleost cell lines expressing endogenous receptors. GPCRs may exhibit distinct signaling properties and physiological functions depending on the cell line or cellular context in which they are expressed, as illustrated above by multiple examples of SUCNR1 signaling [44,45].

In addition, teleost receptors may respond differently from their mammalian counterparts due to lineage-specific gene functional divergence. For many experiments—particularly those involving peptide-activated GPCRs—human-derived peptides are commonly used instead of fish-derived peptides, which may further bias functional interpretation. In contrast, a comparative advantage of studying metabolite-activated GPCRs is that, unlike peptides, metabolites do not differ between fish and mammals, reducing ligand-related variability across species.

Furthermore, functional links between GPCR signaling and physiological traits, including stress coping, nutrient utilization, and aquaculture-relevant economic traits and disease resistance have yet to be demonstrated.

It is also important to consider the basal activity of teleost GPCRs, as some studies have reported relatively high constitutive activity in teleost receptors [12]. In addition, whole-genome duplication in teleosts has resulted in duplicated GPCR paralogs with divergent pharmacology [10,11,149], and the distinct signaling properties and physiological functions among these subtypes warrant further investigation.

Finally, in vivo receptor manipulation studies, including development of exogenous ligands specific to fish GPCRs, gene knockout/knockdown, receptor mutagenesis are scarce, limiting mechanistic understanding.

In summary, despite growing recognition of the importance of metabolite-activated GPCRs, studies in teleosts remain limited. Critical knowledge gaps exist in several areas, including the optimal dietary levels of metabolites as signaling molecules, receptor pharmacology, cell type specificity, and the functional links between GPCR signaling and key physiological traits such as stress response, nutrient utilization, and disease resistance. Teleost-specific factors, including lineage divergence, whole-genome duplication, and basal receptor activity, further complicate interpretation. Addressing these gaps will be essential for understanding the role of metabolite-sensing GPCRs in teleost physiology and for potential applications in aquaculture.

## 6. Conclusions

Metabolite-activated GPCRs represent a central mechanism through which teleosts integrate nutritional, metabolic, and environmental signals to regulate growth, stress responses, and immune function. Despite growing evidence from mammalian systems, key gaps remain in teleosts, including unclear downstream signaling dynamics and insufficient understanding of metabolite doses required to act as signaling molecules rather than nutrients. These axes, such as succinate–SUCNR1, amino acid–GPR142/GPRC6A, and lactate–GPR81, hold clear potential for improving growth efficiency, stress resilience, and disease resistance in aquaculture. Future research should focus on functional validation in vivo, cell-specific signaling characterization, and multi-omics integration to advance mechanistic insight and enable precision aquaculture applications.

## Data Availability

This is a review paper. No new data were created or analyzed in this study.

## References

[1] Bjarnadóttir T.K., Gloriam D.E., Hellstrand S.H., Kristiansson H., Fredriksson R., Schiöth H.B. (2006). Comprehensive repertoire and phylogenetic analysis of the G protein-coupled receptors in human and mouse. Genomics.

[2] Fredriksson R., Lagerström M.C., Lundin L.G., Schiöth H.B. (2003). The G-protein-coupled receptors in the human genome form five main families. Phylogenetic analysis, paralogon groups, and fingerprints. Mol. Pharmacol..

[3] Tao Y.X. (2020). Molecular chaperones and G protein-coupled receptor maturation and pharmacology. Mol. Cell. Endocrinol..

[4] Attwood T.K., Findlay J.B.C. (1994). Fingerprinting G-protein-coupled receptors. Protein Eng. Des. Sel..

[5] Kolakowski L.F. (1994). GCRDb: A G-Protein–Coupled Receptor. Recept. Channels.

[6] Hu G.-M., Mai T.-L., Chen C.-M. (2017). Visualizing the GPCR network: Classification and evolution. Sci. Rep..

[7] Bockaert J., Pin J.P. (1999). Molecular tinkering of G protein-coupled receptors: An evolutionary success. EMBO J..

[8] Husted A.S., Trauelsen M., Rudenko O., Hjorth S.A., Schwartz T.W. (2017). GPCR-mediated signaling of metabolites. Cell Metab..

[9] Lorente J.S., Sokolov A.V., Ferguson G., Schiöth H.B., Hauser A.S., Gloriam D.E. (2025). GPCR drug discovery: New agents, targets and indications. Nat. Rev. Drug Discov..

[10] Lien S., Koop B.F., Sandve S.R., Miller J.R., Kent M.P., Nome T., Hvidsten T.R., Leong J.S., Minkley D.R., Zimin A. (2016). The Atlantic salmon genome provides insights into rediploidization. Nature.

[11] Hou Z.-S., Liu M.-Q., Wen H.-S., Gao Q.-F., Li Z., Yang X.-D., Xiang K.-W., Yang Q., Hu X., Qian M.-Z. (2023). Identification, characterization, and transcription of serotonin receptors in rainbow trout (*Oncorhynchus mykiss*) in response to bacterial infection and salinity changes. Int. J. Biol. Macromol..

[12] Aspiras A.C., Rohner N., Martineau B., Borowsky R.L., Tabin C.J. (2015). Melanocortin 4 receptor mutations contribute to the adaptation of cavefish to nutrient-poor conditions. Proc. Natl. Acad. Sci. USA.

[13] Yang L.K., Zhang Z.R., Wen H.S., Tao Y.X. (2019). Characterization of channel catfish (*Ictalurus punctatus*) melanocortin-3 receptor reveals a potential network in regulation of energy homeostasis. Gen. Comp. Endocrinol..

[14] Ji L.Q., Rao Y.Z., Zhang Y., Chen R., Tao Y.X. (2020). Regulation of melanocortin-1 receptor pharmacology by melanocortin receptor accessory protein 2 in orange-spotted grouper (*Epinephelus coioides*). Gen. Comp. Endocrinol..

[15] Rao Y.Z., Chen R., Zhang Y., Tao Y.X. (2019). Orange-spotted grouper melanocortin-4 receptor: Modulation of signaling by MRAP2. Gen. Comp. Endocrinol..

[16] Oldham W.M., Hamm H.E. (2008). Heterotrimeric G protein activation by G-protein-coupled receptors. Nat. Rev. Mol. Cell Biol..

[17] Tao Y.X., Yuan Z.H., Xie J. (2013). G protein-coupled receptors as regulators of energy homeostasis. Prog. Mol. Biol. Transl. Sci..

[18] Martemyanov K.A., Garcia-Marcos M. (2018). Making useful gadgets with miniaturized G proteins. J. Biol. Chem..

[19] Offermanns S. (2003). G-proteins as transducers in transmembrane signalling. Prog. Biophys. Mol. Biol..

[20] Yang H., Yang L. (2016). Targeting cAMP/PKA pathway for glycemic control and type 2 diabetes therapy. J. Mol. Endocrinol..

[21] Dorsam R.T., Gutkind J.S. (2007). G-protein-coupled receptors and cancer. Nat. Rev. Cancer.

[22] Miller W.E., Lefkowitz R.J. (2001). Expanding roles for β-arrestins as scaffolds and adapters in GPCR signaling and trafficking. Curr. Opin. Cell Biol..

[23] Ritter S.L., Hall R.A. (2009). Fine-tuning of GPCR activity by receptor-interacting proteins. Nat. Rev. Mol. Cell Biol..

[24] Thomsen A.R.B., Plouffe B., Cahill T.J., Shukla A.K., Tarrasch J.T., Dosey A.M., Kahsai A.W., Strachan R.T., Pani B., Mahoney J.P. (2016). GPCR-G protein-β-arrestin super-complex mediates sustained G protein signaling. Cell.

[25] Ahmadian M., Suh J.M., Hah N., Liddle C., Atkins A.R., Downes M., Evans R.M. (2013). PPARγ signaling and metabolism: The good, the bad and the future. Nat. Med..

[26] Mazuy C., Helleboid A., Staels B., Lefebvre P. (2015). Nuclear bile acid signaling through the farnesoid X receptor. Cell. Mol. Life Sci..

[27] Pardella E., Ippolito L., Giannoni E., Chiarugi P. (2022). Nutritional and metabolic signalling through GPCRs. FEBS Lett..

[28] Blad C.C., Tang C., Offermanns S. (2012). G protein-coupled receptors for energy metabolites as new therapeutic targets. Nat. Rev. Drug Discov..

[29] Coogan M., Alston V., Su B., Khalil K., Elaswad A., Khan M., Johnson A., Xing D., Li S.J., Wang J.H. (2022). Improved growth and high inheritance of melanocortin-4 receptor (mc4r) mutation in CRISPR/Cas-9 gene-edited channel catfish, *Ictalurus punctatus*. Mar. Biotechnol..

[30] Metpally R.P.R., Sowdhamini R. (2005). Genome wide survey of G protein-coupled receptors in Tetraodon nigroviridis. BMC Evol. Biol..

[31] Hou Z.S., Wen H.-S. (2022). Neuropeptide Y and melanocortin receptors in fish: Regulators of energy homeostasis. Mar. Life Sci. Technol..

[32] Lethimonier C., Madigou T., Muñoz-Cueto J., Lareyre J.-J., Kah O. (2004). Evolutionary aspects of GnRHs, GnRH neuronal systems and GnRH receptors in teleost fish. Gen. Comp. Endocrinol..

[33] Wang J.-T., Li J.-T., Zhang X.-F., Sun X.-W. (2012). Transcriptome analysis reveals the time of the fourth round of genome duplication in common carp (*Cyprinus carpio*). BMC Genom..

[34] Fernandez-Veledo S., Ceperuelo-Mallafre V., Vendrell J. (2021). Rethinking succinate: An unexpected hormone-like metabolite in energy homeostasis. Trends Endocrinol. Metab..

[35] de Castro Fonseca M., Aguiar C.J., da Rocha Franco J.A., Gingold R.N., Leite M.F. (2016). GPR91: Expanding the frontiers of Krebs cycle intermediates. Cell Commun. Signal..

[36] He W., Miao F.J.P., Lin D.C.H., Schwandner R.T., Wang Z., Gao J., Chen J.L., Tian H., Ling L. (2004). Citric acid cycle intermediates as ligands for orphan G-protein-coupled receptors. Nature.

[37] Grimolizzi F., Arranz L. (2018). Multiple faces of succinate beyond metabolism in blood. Haematologica.

[38] van Diepen J.A., Robben J.H., Hooiveld G.J., Carmone C., Alsady M., Boutens L., Bekkenkamp-Grovenstein M., Hijmans A., Engelke U.F.H., Wevers R.A. (2017). SUCNR1-mediated chemotaxis of macrophages aggravates obesity-induced inflammation and diabetes. Diabetologia.

[39] Serena C., Ceperuelo-Mallafré V., Keiran N., Queipo-Ortuño M.I., Bernal R., Gomez-Huelgas R., Urpi-Sarda M., Sabater M., Pérez-Brocal V., Andrés-Lacueva C. (2018). Elevated circulating levels of succinate in human obesity are linked to specific gut microbiota. ISME J..

[40] De Vadder F., Kovatcheva-Datchary P., Zitoun C., Duchampt A., Bäckhed F., Mithieux G. (2016). Microbiota-produced succinate improves glucose homeostasis via intestinal gluconeogenesis. Cell Metab..

[41] Zhou Z., Yu L., Cao J., Yu J., Lin Z., Hong Y.K., Jiang S., Chen C., Mi Y., Zhang C. (2022). Lactobacillus salivarius promotion of intestinal stem cell activity in hens is associated with succinate-induced mitochondrial energy metabolism. Msystems.

[42] Dai M., Bu S., Miao Z. (2025). Succinate metabolism: Underlying biological mechanisms and emerging therapeutic targets in inflammatory bowel disease. Front. Immunol..

[43] Gilissen J., Jouret F., Pirotte B., Hanson J. (2016). Insight into SUCNR1 (GPR91) structure and function. Pharmacol. Ther..

[44] Trauelsen M., Hiron T.K., Lin D.C.H., Petersen J.E., Breton B., Husted A.S., Hjorth S.A., Inoue A., Frimurer T.M., Bouvier M. (2021). Extracellular succinate hyperpolarizes M2 macrophages through SUCNR1/GPR91-mediated Gq signaling. Cell Rep..

[45] Keiran N., Ceperuelo-Mallafré V., Calvo E., Hernández-Alvarez M.I., Ejarque M., Núñez-Roa C., Horrillo D., Maymó-Masip E., Rodríguez M.M., Fradera R. (2019). SUCNR1 controls an anti-inflammatory program in macrophages to regulate the metabolic response to obesity. Nat. Immunol..

[46] Lu C.Y. (2018). Effects of Dietary Sodium Succinate on Glucose Homeostasis and Nutritional Metabolism of Fish. Master’s Dissertation.

[47] Zhang C., Liu Y., Shi Z., Yao C., Zhang J., Wang Y., Liu J., Mai K., Ai Q. (2024). Effects of dietary succinic acid supplementation on growth performance, digestive ability, intestinal development and immunity of large yellow croaker (*Larimichthys crocea*) larvae. Fish. Shellfish. Immunol..

[48] Liu Y., Cao M., Zhang J., Wang X., Jiang M., Huang F., Cheng K., Dong L., Peng D., Tian J. (2025). Dietary succinic acid mitigates adverse effects of starch high-starch diet in largemouth bass (*Micropterus salmoides*) through gut–liver axis modulation. Fish. Physiol. Biochem..

[49] Yang D., Yang H., Cao Y., Jiang M., Zheng J., Peng B. (2021). Succinate promotes phagocytosis of monocytes/macrophages in teleost fish. Front. Mol. Biosci..

[50] Duan Y., Wang Y., Xiong D., Zhang J. (2019). RNA-seq revealed the signatures of immunity and metabolism in the Litopenaeus vannamei intestine in response to dietary succinate. Fish Shellfish Immunol..

[51] Duan Y., Wang Y., Ding X., Xiong D., Zhang J. (2020). Response of intestine microbiota, digestion, and immunity in Pacific white shrimp Litopenaeus vannamei to dietary succinate. Aquaculture.

[52] Lu Y., Li Y., Bao M., Shang F., Wei R., Liu F., Liu Y., Wang X. (2023). Comparative transcriptome profiling and functional analysis in the blood of tiger puffer (*Takifugu rubripes*) in response to acute hypoxia. Aquac. Rep..

[53] Hamel D., Sanchez M., Duhamel F., Roy O., Honoré J.-C., Noueihed B., Zhou T., Nadeau-Vallée M., Hou X., Lavoie J.-C. (2014). G-protein–coupled receptor 91 and succinate are key contributors in neonatal postcerebral hypoxia-ischemia recovery. Arterioscler. Thromb. Vasc. Biol..

[54] Li T., Hu J., Gao F., Du X., Chen Y., Wu Q. (2017). Transcription factors regulate GPR91-mediated expression of VEGF in hypoxia-induced retinopathy. Sci. Rep..

[55] Huffman K.M., Koves T.R., Hubal M.J., Abouassi H., Beri N., Bateman L.A., Stevens R.D., Ilkayeva O.R., Hoffman E.P., Muoio D.M. (2014). Metabolite signatures of exercise training in human skeletal muscle relate to mitochondrial remodelling and cardiometabolic fitness. Diabetologia.

[56] Reddy A., Bozi L.H.M., Yaghi O.K., Mills E.L., Xiao H., Nicholson H.E., Paschini M., Paulo J.A., Garrity R., Laznik-Bogoslavski D. (2020). pH-gated succinate secretion regulates muscle remodeling in response to exercise. Cell.

[57] Wang T., Xu Y.Q., Yuan Y.X., Xu P.W., Zhang C., Li F., Wang L.N., Yin C., Zhang L., Cai X.C. (2019). Succinate induces skeletal muscle fiber remodeling via SUCNR1 signaling. EMBO Rep..

[58] Stone D.A.J. (2003). Dietary carbohydrate utilization by fish. Rev. Fish. Sci..

[59] Süsens U., Hermans-Borgmeyer I., Urny J., Schaller H.C. (2006). Characterisation and differential expression of two very closely related G-protein-coupled receptors, GPR139 and GPR142, in mouse tissue and during mouse development. Neuropharmacology.

[60] Fredriksson R., Höglund P.J., Gloriam D.E.I., Lagerström M.C., Schiöth H.B. (2003). Seven evolutionarily conserved human rhodopsin G protein-coupled receptors lacking close relatives. FEBS Lett..

[61] Regard J.B., Sato I.T., Coughlin S.R. (2008). Anatomical profiling of G protein-coupled receptor expression. Cell.

[62] Jones K.S., Alimov A.P., Rilo H.L., Jandacek R.J., Woollett L.A., Penberthy W.T. (2008). A high throughput live transparent animal bioassay to identify non-toxic small molecules or genes that regulate vertebrate fat metabolism for obesity drug development. Nutr. Metab..

[63] Lizarzaburu M., Turcotte S., Du X., Duquette J., Fu A., Houze J., Li L., Liu J., Murakoshi M., Oda K. (2012). Discovery and optimization of a novel series of GPR142 agonists for the treatment of type 2 diabetes mellitus. Bioorg. Med. Chem. Lett..

[64] Rudenko O., Shang J., Munk A., Ekberg J.P., Petersen N., Engelstoft M.S., Egerod K.L., Hjorth S.A., Wu M., Feng Y. (2019). The aromatic amino acid sensor GPR142 controls metabolism through balanced regulation of pancreatic and gut hormones. Mol. Metab..

[65] Murakoshi M., Kuwabara H., Nagasaki M., Xiong Y.M., Reagan J.D., Maeda H., Nara F. (2017). Discovery and pharmacological effects of a novel GPR142 antagonist. J. Recept. Signal Transduct..

[66] Ueda Y., Iwakura H., Ensho T., Bando-Shimizu M., Doi A., Matsutani N., Morita S., Inaba H., Ariyasu H., Fukuda N. (2025). Tryptophan-sensing receptor GPR142 expression levels are directly regulated by proinflammatory cytokines in ghrelin-producing cells. FEBS Open Bio.

[67] Liu L.Z., Ma T., Zhou J., Hu Z.L., Zhang X.J., Zhang H.Z., Zeng M., Liu J., Li L., Jiang Y. (2020). Discovery of LY3325656: A GPR142 agonist suitable for clinical testing in human. Bioorg. Med. Chem. Lett..

[68] Machado M., Azeredo R., Domingues A., Fernandez-Boo S., Dias J., Conceição L.E.C., Costas B. (2019). Dietary tryptophan deficiency and its supplementation compromises inflammatory mechanisms and disease resistance in a teleost fish. Sci. Rep..

[69] Machado M., Peixoto D., Santos P., Ricardo A., Duarte I., Carvalho I., Aragão C., Azeredo R., Costas B. (2022). Tryptophan modulatory role in European seabass (*Dicentrarchus labrax*) immune response to acute inflammation under stressful conditions. Int. J. Mol. Sci..

[70] Feng L., Li W., Liu Y., Jiang W.D., Kuang S.Y., Jiang J., Tang L., Wu P., Tang W.N., Zhang Y.A. (2015). Dietary phenylalanine-improved intestinal barrier health in young grass carp (*Ctenopharyngodon idella*) is associated with increased immune status and regulated gene expression of cytokines, tight junction proteins, antioxidant enzymes and related signalling molecules. Fish Shellfish Immunol..

[71] Asencio-Alcudia G., Andree K.B., Giraldez I., Tovar-Ramirez D., Alvarez-González A., Herrera M., Gisbert E. (2019). Stressors due to handling impair gut immunity in meagre (*Argyrosomus regius*): The compensatory role of dietary L-Tryptophan. Front. Physiol..

[72] Kumar P., Pal A.K., Sahu N.P., Jha A.K., Kumar N., Christina L., Priya P. (2018). Dietary L-Tryptophan potentiates non-specific immunity in Labeo rohita fingerlings reared under elevated temperature. J. Therm. Biol..

[73] Lepage O., Tottmar O., Winberg S. (2002). Elevated dietary intake of L-tryptophan counteracts the stress-induced elevation of plasma cortisol in rainbow trout (*Oncorhynchus mykiss*). J. Exp. Biol..

[74] Peixoto D., Carvalho I., Machado M., Aragão C., Costas B., Azeredo R. (2024). Dietary tryptophan intervention counteracts stress-induced transcriptional changes in a teleost fish HPI axis during inflammation. Sci. Rep..

[75] Li W., Feng L., Liu Y., Jiang W.D., Kuang S.Y., Jiang J., Li S.H., Tang L., Zhou X.Q. (2015). Effects of dietary phenylalanine on growth, digestive and brush border enzyme activities and antioxidant capacity in the hepatopancreas and intestine of young grass carp (*Ctenopharyngodon idella*). Aquac. Nutr..

[76] Yi C.G., Liang H.L., Xu G.C., Zhu J., Wang Y.L., Li S.L., Ren M.C., Chen X.R. (2024). Appropriate dietary phenylalanine improved growth, protein metabolism and lipid metabolism, and glycolysis in largemouth bass (*Micropterus salmoides*). Fish Physiol. Biochem..

[77] Rehman S., Gora A.H., Abdelhafiz Y., Dias J., Pierre R., Meynen K., Fernandes J.M.O., Sørensen M., Brugman S., Kiron V. (2023). Potential of algae-derived alginate oligosaccharides and β-glucan to counter inflammation in adult zebrafish intestine. Front. Immunol..

[78] Lv W.Q., Gui J.F. (2020). Stress coping strategy and its application in stress resistance breeding in fish. J. Fish. China.

[79] Huntingford F.A., Andrew G., Mackenzie S., Morera D., Coyle S.M., Pilarczyk M., Kadri S. (2010). Coping strategies in a strongly schooling fish, the common carp *Cyprinus carpio*. J. Fish Biol..

[80] Øverli Ø., Sørensen C., Nilsson G.E. (2006). Behavioral indicators of stress-coping style in rainbow trout: Do males and females react differently to novelty?. Physiol. Behav..

[81] Martins C.I.M., Schrama J.W., Verreth J.A.J. (2005). The consistency of individual differences in growth, feed efficiency and feeding behaviour in African catfish *Clarias gariepinus* (Burchell 1822) housed individually. Aquac. Res..

[82] Øverli Ø., Sørensen C., Pulman K.G.T., Pottinger T.G., Korzan W., Summers C.H., Nilsson G.E. (2007). Evolutionary background for stress-coping styles: Relationships between physiological, behavioral, and cognitive traits in non-mammalian vertebrates. Neurosci. Biobehav. Rev..

[83] Höglund E., Gjøen H.M., Pottinger T.G., Øverli Ø. (2008). Parental stress-coping styles affect the behaviour of rainbow trout *Oncorhynchus mykiss* at early developmental stages. J. Fish Biol..

[84] Silva P.I.M., Martins C.I.M., Engrola S., Marino G., Øverli Ø., Conceição L.E.C. (2010). Individual differences in cortisol levels and behaviour of Senegalese sole (*Solea senegalensis*) juveniles: Evidence for coping styles. Appl. Anim. Behav. Sci..

[85] Castanheira M.F., Herrera M., Costas B., Conceição L.E., Martins C.I. (2013). Linking cortisol responsiveness and aggressive behaviour in gilthead seabream Sparus aurata: Indication of divergent coping styles. Appl. Anim. Behav. Sci..

[86] Trenzado C.E., Carrick T.R., Pottinger T.G. (2003). Divergence of endocrine and metabolic responses to stress in two rainbow trout lines selected for differing cortisol responsiveness to stress. Gen. Comp. Endocrinol..

[87] Schjolden J., Pulman K.G.T., Pottinger T.G., Tottmar O., Winberg S. (2006). Serotonergic characteristics of rainbow trout divergent in stress responsiveness. Physiol. Behav..

[88] Øverli Ø., Winberg S., Pottinger T.G. (2005). Behavioral and neuroendocrine correlates of selection for stress responsiveness in rainbow trout—A review. Integr. Comp. Biol..

[89] Vindas M.A., Johansen I.B., Folkedal O., Höglund E., Gorissen M., Flik G., Kristiansen T.S., Øverli Ø. (2016). Brain serotonergic activation in growth-stunted farmed salmon: Adaption versus pathology. R. Soc. Open Sci..

[90] Cabanillas-Gámez M., López L.M., Galaviz M.A., True C.D., Bardullas U. (2018). Effect of L-tryptophan supplemented diets on serotonergic system and plasma cortisol in Totoaba macdonaldi (Gilbert, 1890) juvenile exposed to acute stress by handling and hypoxia. Aquac. Res..

[91] Laranja J.L.Q., Quinitio E.T., Catacutan M.R., Coloso R.M. (2010). Effects of dietary L-tryptophan on the agonistic behavior, growth and survival of juvenile mud crab Scylla serrata. Aquaculture.

[92] Morandini L., Ramallo M.R., Moreira R.G., Höcht C., Somoza G.M., Silva A., Pandolfi M. (2015). Serotonergic outcome, stress and sexual steroid hormones, and growth in a South American cichlid fish fed with an L-tryptophan enriched diet. Gen. Comp. Endocrinol..

[93] Moon T.W. (2001). Glucose intolerance in teleost fish: Fact or fiction? Comparative biochemistry and physiology. Part B Biochem. Mol. Biol..

[94] Navarro I., Rojas P., Capilla E., Albalat A., Castillo J., Montserrat N., Codina M., Gutiérrez J.J.F.P. (2002). Insights into insulin and glucagon responses in fish. Fish Physiol. Biochem..

[95] Riddle M.R., Aspiras A.C., Gaudenz K., Peuß R., Sung J.Y., Martineau B., Peavey M., Box A.C., Tabin J.A., McGaugh S. (2018). Insulin resistance in cavefish as an adaptation to a nutrient-limited environment. Nature.

[96] Xiao K., Jia X.C., Qiang W., Chang L., Liu W., Zhang D. (2024). Tryptophan supplements in high-carbohydrate diets by improving insulin response and glucose transport through PI3K-AKT-GLUT2 pathways in blunt snout bream (*Megalobrama amblycephala*). J. Nutr. Biochem..

[97] Du W., Jiang S., Yin S., Wang R., Zhang C., Yin B.C., Li J., Li L., Qi N., Zhou Y. (2024). The microbiota-dependent tryptophan metabolite alleviates high-fat diet–induced insulin resistance through the hepatic AhR/TSC2/mTORC1 axis. Proc. Natl. Acad. Sci. USA.

[98] Breum L., Rasmussen M.H., Hilsted J., Fernstrom J.D. (2003). Twenty-four–hour plasma tryptophan concentrations and ratios are below normal in obese subjects and are not normalized by substantial weight reduction. Am. J. Clin. Nutr..

[99] Itoh Y., Kawamata Y., Harada M., Kobayashi M., Fujii R., Fukusumi S., Ogi K., Hosoya M., Tanaka Y., Uejima H. (2003). Free fatty acids regulate insulin secretion from pancreatic β cells through GPR40. Nature.

[100] Wellendorph P., Hansen K.B., Balsgaard A., Greenwood J.R., Egebjerg J., Brauner-Osborne H. (2005). Deorphanization of GPRC6A: A promiscuous L-α-amino acid receptor with preference for basic amino acids. Mol. Pharmacol..

[101] Kuang D., Yao Y., Lam J., Tsushima R.G., Hampson D.R. (2005). Cloning and characterization of a family C orphan G-protein coupled receptor. J. Neurochem..

[102] Mizokami A., Otani T., Mukai S., Hirata M. (2024). Roles of Nutrition-Sensing Receptor GPRC6A in Energy Metabolism and Oral Inflammatory Diseases. Curr. Oral Health Rep..

[103] Jørgensen C.V., Bräuner-Osborne H. (2020). Pharmacology and physiological function of the orphan GPRC6A receptor. Basic Clin. Pharmacol. Toxicol..

[104] Christiansen B., Hansen K.B., Wellendorph P., Bräuner-Osborne H. (2007). Pharmacological characterization of mouse GPRC6A, an L-α-amino-acid receptor modulated by divalent cations. Br. J. Pharmacol..

[105] Pi M., Faber P., Ekema G., Jackson P.D., Ting A., Wang N., Fontilla-Poole M., Mays R.W., Brunden K.R., Harrington J.J. (2005). Identification of a novel extracellular cation-sensing G-protein-coupled receptor. J. Biol. Chem..

[106] Pi M., Parrill A.L., Quarles L.D. (2010). GPRC6A mediates the non-genomic effects of steroids. J. Biol. Chem..

[107] Jacobsen S.E., Ammendrup-Johnsen I., Jansen A.M., Gether U., Madsen K.L., Bräuner-Osborne H. (2017). The GPRC6A receptor displays constitutive internalization and sorting to the slow recycling pathway. J. Biol. Chem..

[108] Conigrave A.D., Hampson D.R. (2006). Broad-spectrum L-amino acid sensing by class 3 G-protein-coupled receptors. Trends Endocrinol. Metab..

[109] Oya M., Kitaguchi T., Pais R., Reimann F., Gribble F., Tsuboi T. (2013). The G protein-coupled receptor family C group 6 subtype A (GPRC6A) receptor is involved in amino acid-induced glucagon-like peptide-1 secretion from GLUTag cells. J. Biol. Chem..

[110] Rueda P., Harley E., Lu Y., Stewart G.D., Fabb S., Diepenhorst N., Cremers B., Rouillon M.H., Wehrle I., Geant A. (2016). Murine GPRC6A mediates cellular responses to L-amino acids, but not osteocalcin variants. PLoS ONE.

[111] Alamshah A., McGavigan A.K., Spreckley E., Kinsey-Jones J.S., Amin A., Tough I.R., O’hara H.C., Moolla A., Banks K., France R. (2016). L-Arginine promotes gut hormone release and reduces food intake in rodents. Diabetes Obes. Metab..

[112] Clemmensen C., Jørgensen C.V., Smajilovic S., Bräuner-Osborne H. (2017). Robust GLP-1 secretion by basic L-amino acids does not require the GPRC6A receptor. Diabetes Obes. Metab..

[113] Wellendorph P., Johansen L.D., Bräuner-Osborne H. (2009). Molecular pharmacology of promiscuous seven transmembrane receptors sensing organic nutrients. Mol. Pharmacol..

[114] Wauson E.M., Lorente-Rodriguez A., Cobb M.H. (2013). Minireview: Nutrient sensing by G protein-coupled receptors. Mol. Endocrinol..

[115] Gong T., Liu L., Jiang W., Zhou R. (2020). DAMP-sensing receptors in sterile inflammation and inflammatory diseases. Nat. Rev. Immunol..

[116] Rossol M., Pierer M., Raulien N., Quandt D., Meusch U., Rothe K., Schubert K., Schöneberg T., Schaefer M., Krügel U. (2012). Extracellular Ca2+ is a danger signal activating the NLRP3 inflammasome through G protein-coupled calcium sensing receptors. Nat. Commun..

[117] Lee G.S., Subramanian N., Kim A.I., Aksentijevich I., Goldbach-Mansky R., Sacks D.B., Germain R.N., Kastner D.L., Chae J.J. (2012). The calcium-sensing receptor regulates the NLRP3 inflammasome through Ca2+ and cAMP. Nature.

[118] Wang L., Wu J., Wang C.A., Li J., Zhao Z., Luo L., Du X., Xu Q. (2017). Dietary arginine requirement of juvenile hybrid sturgeon (*Acipenser schrenckii*♀ × *Acipenser baerii*♂). Aquac. Res..

[119] Chen Q., Zhao H., Huang Y., Cao J., Wang G., Sun Y., Li Y. (2016). Effects of dietary arginine levels on growth performance, body composition, serum biochemical indices and resistance ability against ammonia-nitrogen stress in juvenile yellow catfish (*Pelteobagrus fulvidraco*). Anim. Nutr..

[120] Chen G., Liu Y., Jiang J., Jiang W., Kuang S.Y., Tang L., Tang W., Zhang Y.A., Zhou X.Q., Feng L. (2015). Effect of dietary arginine on the immune response and gene expression in head kidney and spleen following infection of Jian carp with Aeromonas hydrophila. Fish Shellfish Immunol..

[121] Calo J., Blanco A.M., Comesaña S., Conde-Sieira M., Morais S., Soengas J.L. (2021). First evidence for the presence of amino acid sensing mechanisms in the fish gastrointestinal tract. Sci. Rep..

[122] Calo J., Soengas J.L., Pastor J.J., Blanco A.M., Morais S. (2023). Evidence of gastrointestinal sensing and gut-brain communication in rainbow trout (*Oncorhynchus mykiss*) in response to the aqueous extract of fishmeal and its free amino acid fraction. Aquaculture.

[123] Canè S., Geiger R., Bronte V. (2025). The roles of arginases and arginine in immunity. Nat. Rev. Immunol..

[124] Geiger R., Rieckmann J.C., Wolf T., Basso C., Feng Y., Fuhrer T., Kogadeeva M., Picotti P., Meissner F., Mann M. (2016). L-arginine modulates T cell metabolism and enhances survival and anti-tumor activity. Cell.

[125] Irwin D.M., Mojsov S. (2018). Diversification of the functions of proglucagon and glucagon receptor genes in fish. Gen. Comp. Endocrinol..

[126] Ahmed K., Tunaru S., Tang C., Müller M., Gille A., Sassmann A., Hanson J., Offermanns S. (2010). An autocrine lactate loop mediates insulin-dependent inhibition of lipolysis through GPR81. Cell Metab..

[127] Brown T.P., Ganapathy V. (2020). Lactate/GPR81 signaling and proton motive force in cancer: Role in angiogenesis, immune escape, nutrition, and Warburg phenomenon. Pharmacol. Ther..

[128] Colucci A.C.M., Tassinari I.D.Á., da Silveira Loss E., de Fraga L.S. (2023). History and function of the lactate receptor GPR81/HCAR1 in the brain: A putative therapeutic target for the treatment of cerebral ischemia. Neuroscience.

[129] Offermanns S., Colletti S.L., Lovenberg T.W., Semple G., Wise A., IJzerman A.P. (2011). International Union of Basic and Clinical Pharmacology. LXXXII: Nomenclature and classification of hydroxy-carboxylic acid receptors (GPR81, GPR109A, and GPR109B). Pharmacol. Rev..

[130] Liu C., Wu J., Zhu J., Kuei C., Yu J., Shelton J., Sutton S.W., Li X., Yun S.J., Mirzadegan T. (2009). Lactate inhibits lipolysis in fat cells through activation of an orphan G-protein-coupled receptor, GPR81. J. Biol. Chem..

[131] Ge H., Weiszmann J., Reagan J.D., Gupte J., Baribault H., Gyuris T., Chen J.L., Tian H., Li Y. (2008). Elucidation of signaling and functional activities of an orphan GPCR, GPR81. J. Lipid Res..

[132] Lee Y.-S., Kim T.-Y., Kim Y.B., Lee S.-H., Kim S., Kang S.W., Yang J.-Y., Baek I.-J., Sung Y.H., Park Y.-Y. (2018). Microbiota-derived lactate accelerates intestinal stem-cell-mediated epithelial development. Cell Host Microbe.

[133] Fang H., Anhê F.F., Zada D.K., Barra N.G., McAlpin B.T., Wylie R., Berthiaume L., Audet-Walsh É., O’Dwyer C., Ghorbani P. (2025). Gut substrate trap of D-lactate from microbiota improves blood glucose and fatty liver disease in obese mice. Cell Metab..

[134] Briquet M., Rocher A.-B., Alessandri M., Rosenberg N., de Castro Abrantes H., Wellbourne-Wood J., Schmuziger C., Ginet V., Puyal J., Pralong E. (2022). Activation of lactate receptor HCAR1 down-modulates neuronal activity in rodent and human brain tissue. J. Cereb. Blood Flow Metab..

[135] Hoque R., Farooq A., Ghani A., Gorelick F., Mehal W.Z. (2014). Lactate reduces liver and pancreatic injury in toll-like receptor–and inflammasome-mediated inflammation via GPR81-mediated suppression of innate immunity. Gastroenterology.

[136] Lerch M.M., Conwell D.L., Mayerle J. (2014). The anti-inflammasome effect of lactate and the lactate GPR81-receptor in pancreatic and liver inflammation. Gastroenterology.

[137] Li R.X., Zhou W.H., Ren J., Wang J., Qiao F., Zhang M.L., Du Z.Y. (2022). Dietary sodium lactate promotes protein and lipid deposition through increasing energy supply from glycolysis in Nile tilapia (*Oreochromis niloticus*). Aquaculture.

[138] Abdel-Aziz M.F.A., El Basuini M.F., Sadek M.F., Elokaby M.A., El-Dakar A.Y., Metwally M.M.M., Shehab A., Mabrok M., Abdel Rahman A.N. (2024). Unchanged water stress induces growth retardation, histopathological alterations, and antioxidant-immune disruptions in Oreochromis niloticus: The promising role of dietary organic acids. Aquac. Int..

[139] Hoseini S.M., Rajabiesterabadi H., Abbasi M., Khosraviani K., Hoseinifar S.H., Van Doan H. (2022). Modulation of humoral immunological and antioxidant responses and gut bacterial community and gene expression in rainbow trout, *Oncorhynchus mykiss*, by dietary lactic acid supplementation. Fish Shellfish Immunol..

[140] Kuei C., Yu J., Zhu J., Wu J., Zhang L., Shih A., Mirzadegan T., Lovenberg T.W., Liu C. (2011). Study of GPR81, the lactate receptor, from distant species identifies residues and motifs critical for GPR81 functions. Mol. Pharmacol..

[141] Sun J., Ye X., Xie M., Ye J. (2016). Induction of triglyceride accumulation and mitochondrial maintenance in muscle cells by lactate. Sci. Rep..

[142] Longhitano L., Forte S., Orlando L., Grasso S., Barbato A., Vicario N., Parenti R., Fontana P., Amorini A.M., Lazzarino G. (2022). The crosstalk between GPR81/IGFBP6 promotes breast cancer progression by modulating lactate metabolism and oxidative stress. Antioxidants.

[143] Talarico G.G., Thoral E., Farhat E., Teulier L., Mennigen J.A., Weber J.-M. (2023). Lactate signaling and fuel selection in rainbow trout: Mobilization of energy reserves. Am. J. Physiol. Regul. Integr. Comp. Physiol..

[144] Rivero-Müller A., Chou Y.Y., Ji I., Lajic S., Hanyaloglu A.C., Jonas K., Rahman N., Ji T.H., Huhtaniemi I. (2010). Rescue of defective G protein–coupled receptor function in vivo by intermolecular cooperation. Proc. Natl. Acad. Sci. USA.

[145] Catt K.J., Dufau M.L. (1973). Spare gonadotrophin receptors in rat testis. Nat. New Biol..

[146] Hoseini S.M., Yousefi M., Afzali-Kordmahalleh A., Pagheh E., Taheri Mirghaed A. (2023). Effects of dietary lactic acid supplementation on the activity of digestive and antioxidant enzymes, gene expressions, and bacterial communities in the intestine of common carp, *Cyprinus carpio*. Animals.

[147] Nguyen L., Salem S.M.R., Salze G.P., Dinh H., Davis D.A. (2019). Tryptophan requirement in semi-purified diets of juvenile Nile tilapia Oreochromis niloticus. Aquaculture.

[148] Ahmed I., Ahmad I., Malla B.A. (2024). Effects of dietary tryptophan levels on growth performance, plasma profile, intestinal antioxidant capacity and growth related genes in rainbow trout (*Oncorhynchus mykiss*) fingerlings. Aquaculture.

[149] Ji R.-L., Liu T., Hou Z.-S., Wen H.-S., Tao Y.-X. (2023). Divergent pharmacology and biased signaling of the four melanocortin-4 receptor isoforms in rainbow trout (*Oncorhynchus mykiss*). Biomolecules.

